# Ecotoxicological Impacts of Heavy Metals on Medicinal Plant Quality and Rhizosphere Microbial Communities

**DOI:** 10.3390/plants14203214

**Published:** 2025-10-19

**Authors:** Hexigeduleng Bao, Yu Wang, Hainan Bao, Feijuan Wang, Qiong Jiang, Xiaoqi He, Hua Li, Yanfei Ding, Cheng Zhu

**Affiliations:** 1 Key Laboratory of Specialty Agri-Product Quality and Hazard Controlling Technology of Zhejiang Province, College of Life Science, China Jiliang University, Hangzhou 310018, China; wangyu90203@163.com (Y.W.); 13848880640@163.com (H.B.); wfj0311@cjlu.edu.cn (F.W.); qjiang@cjlu.edu.cn (Q.J.); dingyanfei@cjlu.edu.cn (Y.D.); pzhch@cjlu.edu.cn (C.Z.); 2 College of Engineering, Nanjing Agricultural University, Nanjing 210031, China; lihua@njau.edu.cn; 3 Ningbo Industrial Internet Institute Co., Ltd., Ningbo 315000, China; hexiaoqi@niii.com

**Keywords:** heavy metal pollution, medicinal plants, rhizosphere microorganisms, impact mechanisms, ecological safety

## Abstract

With the rapid expansion of industrial activities, the accumulation of heavy metals in the environment has become a serious threat to ecological security and public health. Rhizosphere microorganisms play a crucial role in supporting the growth and quality of medicinal plants by facilitating nutrient uptake and regulating hormonal balance. However, medicinal plants can absorb heavy metals from contaminated soils during growth, resulting in toxic metal accumulation in plant tissues and reduced efficacy of active compounds. At the same time, excessive heavy metal levels suppress rhizosphere microbial growth and activity, disrupt community structure and function, and weaken their beneficial interactions with plants. These processes collectively lead to soil fertility decline, hindered plant development, and compromised safety and quality of medicinal materials. This review systematically summarizes the mechanisms by which heavy metals affect medicinal plants and their rhizosphere microbiota, and highlights that future research should focus on elucidating these interactions, developing advanced remediation technologies, and establishing comprehensive monitoring systems for the quality and safety of medicinal plants, thereby providing a scientific basis for their safe utilization and quality improvement.

## 1. Introduction

### 1.1. Sources and Characteristics of Heavy Metal Pollution

In recent years, the issue of heavy metal pollution has become increasingly severe due to a variety of activities, including industrial emissions, agricultural practices, mining, smelting, and transportation [1]. These pollution sources introduce heavy metals into water bodies, the atmosphere, and soil through wastewater, exhaust gases, and solid waste, exacerbating environmental contamination and posing significant threats to ecosystems and human health [2].

The pollution of heavy metals is characterized by its widespread sources and long-lasting presence, with most heavy metals unable to be readily degraded through natural biological processes [3,4]. Studies have shown that heavy metals can be transmitted through the food chain and accumulate in living organisms, resulting in concentrations significantly higher than those found in the surrounding environment [5]. Additionally, heavy metal pollution has long-term persistence and strong mobility [6]. Prolonged exposure or high concentrations of heavy metals pose potential risks to human health. Given these challenges, the cultivation and management of crops, including medicinal plants, must strictly control heavy metal content. Heavy metals not only inhibit plant growth and active compound synthesis but also alter microbial community structures, reduce biodiversity, and impair critical ecological functions such as nutrient cycling and pathogen resistance. These disruptions underscore the urgent need to understand the toxicological mechanisms and ecological consequences of heavy metals on the plant-microbe system.

### 1.2. Importance and Extensive Applications of Medicinal Plants

Medicinal plants hold significant medical and health value globally and are widely applied in the prevention and treatment of various diseases. Many medicinal plants are also classified as “food-medicine homologous plants”. The uniqueness of medicinal plants lies in their comprehensive effects, which focus not only on disease treatment but also on disease prevention and the enhancement of individual health. Representative medicinal plants such as *Panax ginseng*, *Dendrobium officinale*, and *Ginkgo biloba* are extensively used in health supplements and pharmaceuticals, with ongoing advancements in the research and development of their active compounds. With continued scientific exploration, increasing numbers of bioactive components from medicinal plants have been identified to possess anti-inflammatory [7], antioxidant [8], and anti-tumor [9] activities, solidifying their place in modern drug development. As global health governance becomes increasingly critical, the role of medicinal plants is becoming more prominent, providing invaluable resources for improving human health and preventing diseases.

### 1.3. Overview and Roles of Rhizosphere Microorganisms

The rhizosphere, the region surrounding plant roots, is the primary site of interaction between plants and microorganisms. Within and around the rhizosphere of medicinal plants, a large number of microorganisms, including bacteria, fungi, and actinomycetes, thrive. These microorganisms form intricate interactions with plant roots, creating unique microbial communities [10]. Through these interactions, they establish a vital network linking medicinal plants and soil [11], which plays a crucial role in maintaining the quality of medicinal plants and the stability of ecosystems. However, current studies often lack comprehensive evaluations of the toxicological impacts of heavy metals on rhizosphere microbial communities and their cascading effects on medicinal plant quality. Specifically, the relationships between heavy metal concentration, microbial diversity, and plant active compound synthesis remain largely unclear. This review seeks to address these gaps by focusing on the ecological and toxicological mechanisms underlying these interactions.

Microorganisms in the rhizosphere play an essential role in material cycling and energy transformation within the soil. By facilitating the biotransformation and accumulation of trace elements in the rhizosphere, microorganisms significantly enhance the ability of medicinal plant roots to absorb critical nutrients [12]. Li et al. [13] reported that rhizosphere microorganisms release chemical substances that promote nutrient uptake and transport, thereby increasing the accumulation of active compounds in medicinal plants. This process serves as a key driver for the growth and development of medicinal plants and directly determines the final quality and efficacy of medicinal materials. Rhizosphere microorganisms play a pivotal role in regulating the bioavailability of nutrients and mitigating the toxicity of heavy metals, ensuring plant health and sustainable growth [14,15]. They optimize the growth environment for medicinal plants, thereby promoting the synthesis and accumulation of bioactive compounds, which are critical for determining the quality of medicinal materials. Therefore, a deep understanding and effective utilization of the functional roles of rhizosphere microbial communities have significant practical value and application potential for improving the yield and pharmacological efficacy of medicinal plants.

Table 1 summarizes the mechanisms and roles of rhizosphere microorganisms in medicinal plants. Rhizosphere microorganisms play critical roles in supporting medicinal plants through diverse mechanisms. They regulate root exudates, altering root morphology and microbial colonization, and enhance secondary metabolite synthesis via pathways influenced by nitrogen-fixing bacteria. Microorganisms like *Bacillus* improve soil enzyme activity and antioxidant responses, boosting stress resistance. They aid nutrient absorption by mineralizing phosphorus and producing siderophores, while actinomycetes enhance disease resistance by decomposing organic matter. For heavy metal detoxification, microorganisms chelate or adsorb metals, reducing their uptake by plants. They also regulate plant hormone levels, promote secondary metabolite synthesis, and facilitate biodegradation and bioaccumulation for environmental remediation. Microbial products such as indole-3-acetic acid (IAA) promote plant growth, while extracellular polymeric substances (EPS) improve soil structure and water retention, enhancing plant resilience and productivity. Collectively, these functions underscore the essential role of rhizosphere microorganisms in maintaining medicinal plant health and environmental sustainability (Table 1).

## 2. Comprehensive Effects of Heavy Metals on the Quality of Medicinal Plants

### 2.1. Effects of Heavy Metals on the Phenotypic Characteristics of Medicinal Plants

Heavy metal pollution, such as cadmium (Cd), arsenic (As), and lead (Pb), exerts widespread effects on the growth and development of medicinal plants. These metals inhibit cell division and expansion, resulting in stunted plant growth and poorly developed root systems. Additionally, they disrupt chlorophyll synthesis, causing chlorosis or whitening of medicinal plant leaves [34,35]. In the root systems, the accumulation of heavy metals severely hinders the absorption of water and essential nutrients, leading to root tip degeneration and restricted growth [36]. Further studies have shown that heavy metals can also affect plant reproduction by reducing pollen viability and inhibiting seed maturation, ultimately resulting in decreased reproductive capacity [37].

### 2.2. Mechanisms of Heavy Metal Effects on the Active Compounds in Medicinal Plants

#### 2.2.1. Characteristics and Classification of Active Compounds in Medicinal Plants

Active compounds in medicinal plants refer to naturally occurring substances within medicinal materials that reflect their physiological and biological functions. These compounds typically possess highly specific pharmacological activities and interact with particular biological targets in the human body, such as molecules and cellular structures, to exert therapeutic effects. There are many types of active compounds in medicinal plants, including but not limited to polysaccharides, flavonoids, alkaloids, saponins, and phenolic acids [38]. These compounds exhibit diverse pharmacological activities. For example, polysaccharides in medicinal plants have been shown to significantly enhance immunity and antiviral capacity [39,40], making them valuable in promoting physical health and as adjuncts for anti-tumor therapies. Flavonoids, recognized for their strong antioxidant properties [41], effectively scavenge free radicals, reducing the risk of cardiovascular diseases. These compounds are widely used in the prevention and treatment of cardiovascular and cerebrovascular conditions. Alkaloid compounds primarily act by regulating nervous system functions [42] and are commonly applied to alleviate pain and treat cardiovascular-related disorders. Saponins, through their ability to modulate immune system functions [43], can enhance the body’s resistance to disease while exhibiting significant anti-inflammatory effects. Phenolic acids, with their antioxidant and anti-inflammatory properties [44], contribute to the prevention and treatment of various diseases, improving overall health conditions. The diversity and complexity of these active compounds, along with their mechanisms of action, are central to medicinal plant research. They offer immense research and application potential in modern medicine, serving as valuable resources for new therapeutic developments.

#### 2.2.2. Effects of Heavy Metals on the Synthesis and Accumulation of Active Compounds in Medicinal Plants

**Figure 1:**
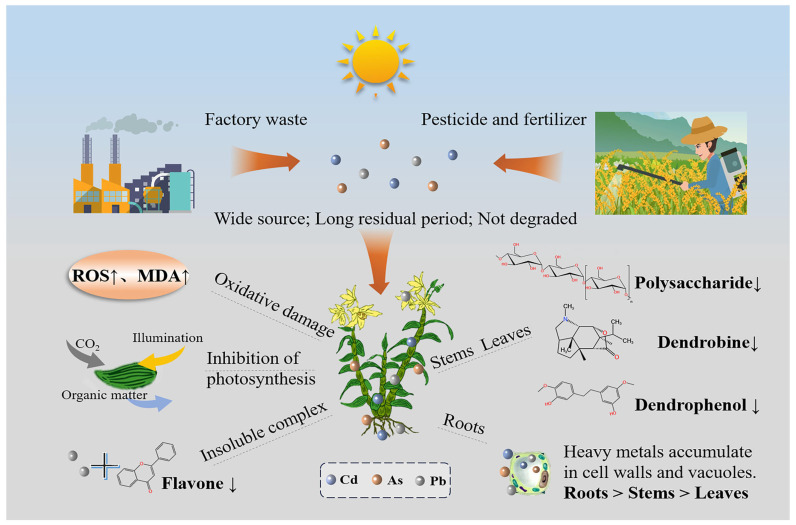
Influence of heavy metals on the quality of medicinal plants. Medicinal plants can uptake heavy metals from contaminated soils during growth, leading to their accumulation in plant tissues, oxidative stress, and disruption of metabolic pathways, which significantly compromises the efficacy of active compounds.

Heavy metals are widely present in the environment and generally exhibit negative effects on the synthesis and accumulation of active compounds in medicinal plants, as illustrated in Figure 1.

The *Pharmacopoeia of the People’s Republic of China* (Volume IV) provides the regulatory basis for maximum permissible limits of heavy metal residues in medicinal plants. When these thresholds are exceeded, the levels of active compounds may decrease, leading to a decline in quality and compromising both therapeutic efficacy and safety. To place these values within a wider regulatory landscape, we also compared them with limits recommended by the World Health Organization (WHO) and with the ICH Q3D (R2) guideline adopted by the United States Pharmacopeia (USP) and the European Medicines Agency (EMA), as summarized in Table 2.

These differences illustrate distinct regulatory philosophies: the *Pharmacopoeia of the People’s Republic of China* and WHO express limits as concentration-based thresholds (mg·kg^−1^) for herbal raw materials, while ICH Q3D/USP/EMA apply risk-based permitted daily exposures (µg/day) for finished drug products. This lack of harmonization highlights the importance of product-specific risk assessment when applying international standards to medicinal plant quality control and clinical safety. Different heavy metals, however, have distinct effects on medicinal plants.

(1) Cadmium (Cd):

Cd primarily inhibits the activity of phenylalanine ammonia-lyase (PAL), an enzyme of the phenylpropanoid pathway, reducing the synthesis of secondary metabolites such as alkaloids and saponins [49]. Under low-concentration Cd treatment (5 mg·L^−1^, 10 mg·L^−1^), the content of low-molecular-weight organic acids, except oxalic acid, significantly increased in the root exudates of *Lonicera japonica*, along with an increase in the total amino acid secretion. Under high-concentration Cd treatment (25 mg·L^−1^, 50 mg·L^−1^), the content of all components in the root exudates significantly decreased, resulting in a reduction in Cd absorption capacity by *Lonicera japonica*. Low-concentration Cd treatment significantly promoted the accumulation of organic acid active components in the leaves, and although their content decreased with increasing Cd concentration, it remained higher than that of the control group [50]. Compared with the control, Cd stress significantly reduced the content of water-soluble phenolic acids, such as caffeic acid and rosmarinic acid, in the leaves of *Salvia miltiorrhiza*, while increasing the content of protocatechuic acid [51]. The contents of salvianolic acid, protocatechualdehyde, and salvianolic acid B also decreased, but the changes were not significant. In the roots, the levels of all six phenolic acids decreased, with rosmarinic acid showing the most significant change. In *Salvia miltiorrhiza* under Cd stress, the contents of lipid-soluble tanshinones, including dihydrotanshinone, tanshinone I, and cryptotanshinone, in the roots significantly decreased, whereas the content of tanshinone IIA remained unchanged. In the leaves of *Salvia miltiorrhiza*, the activities of key enzymes involved in the synthesis of water-soluble phenolic acids, such as PAL and tyrosine aminotransferase (TAT), were significantly reduced, but the activities of cinnamate 4-hydroxylase (C4H) and 4-coumarate-CoA ligase (4CL) significantly increased. These results indicate that Cd stress can reduce both the yield and quality of *Salvia miltiorrhiza* [51]. Zhang et al. [52] reported that under Cd stress, *Chrysanthemum indicum* showed a decline in medicinal quality. The study comprehensively examined six active components, including linarin, total flavonoids, quercetin, apigenin, robinin, and luteolin. Among these, the quality of *Dendranthema indicum* medicinal materials, as indicated by linarin, total flavonoids, apigenin, quercetin, and luteolin, was significantly lower under different concentrations (5–100 mg·kg^−1^) of Cd stress than those in the control, resulting in a decline in the overall quality of the medicinal materials. However, the accumulation of robinin showed both promotive and inhibitory effects depending on the concentration of Cd stress [52]. Transcriptome sequencing and bioinformatics analysis were conducted to investigate the transcriptomic profiles of *Plantago asiatica* seedlings under two Cd treatments (0 mg·kg^−1^ and 50 mg·kg^−1^). KEGG pathway enrichment analysis revealed that numerous differentially expressed genes (DEGs) were enriched in metabolic pathways such as sucrose and starch metabolism, plant hormone signal transduction, pentose and glucuronate interconversions, and phenylpropanoid biosynthesis. Further analysis indicated that genes associated with flavonoids, sucrose, soluble sugars, peroxidase, lignin, and pectin—substances related to stress resistance—likely play crucial roles in the response of *Plantago asiatica* to Cd stress [53]. There were 78 metabolites showing significant differences between the Cd-treated group and control group, including 31 up-regulated and 47 down-regulated metabolites, mainly carbohydrates and carboxylic acids. The differential metabolites were mainly enriched in 20 pathways, including three glucose metabolism pathways and three lipid metabolism pathways. Nevertheless, studies on the effects of Cd on a specific active compound or particular active components in medicinal plants are still quite limited.

(2) Arsenic (As):

As is a highly cytotoxic element that disrupts normal cellular functions. Kamali et al. [54] investigated the effects of As on the metabolism of *Thymus kotschyanus* seedlings and found that As exposure (10 mg·L^−1^) significantly impacted the metabolism by reducing photosynthetic pigments, including chlorophylls and carotenoids, and altering protein concentrations. It also triggered oxidative stress, as well as PAL enzyme activities of the phenylpropanoid pathway (which includes metabolites such as flavonoids, lignin, and phenolic acids). Additionally, As exposure disrupted secondary metabolite synthesis, particularly affecting terpenoid metabolism. However, methyl jasmonate (MJ, 10 µM) supplementation alleviated these adverse effects by upregulating key metabolic pathways, enhancing γ-terpinene synthase (.) gene expression, increasing proline and soluble phenol accumulation, and stimulating enzymatic antioxidant responses. Two cytochrome P450 monooxygenases, including *CYP71D178* and *CYP71D180*, were upregulated in response to As and MJ. These findings highlight the detrimental effects of As on both primary and secondary plant metabolism, alongside MJ’s potential to mitigate As toxicity through metabolic regulation. Chen et al. [55] found that As in *Panax notoginseng* roots primarily accumulates in epidermal tissues and tends to migrate toward the vascular bundles, with vacuolar compartments serving as the main sites of As accumulation. Nazir et al. [56] reported that As inhibits photosynthesis and ROS production, while also interfering with critical biological processes such as carbohydrate metabolism and chlorophyll synthesis. Yuan et al. [57] discovered that As can impede plant growth and development, with inorganic As (NaAsO_2_) being significantly more toxic to plants than organic As (dimethyl arsenic). Low concentrations of inorganic arsenic (10–20 mg·L^−1^) and 10–50 mg·L^−1^ organic arsenic had a stimulating effect on the growth of honeysuckle plants, while the antioxidant system in honeysuckle plants was damaged under high concentrations of inorganic arsenic (20–40 mg·L^−1^) and organic arsenic (50–70 mg·L^−1^). Inorganic As further reduces the synthesis of secondary metabolites, exerting greater adverse effects on the accumulation of active compounds [56].

(3) Lead (Pb):

Pb disrupts the balance of essential elements in plants through antagonistic effects, causing nutrient stress and indirectly affecting plant growth and development [58]. Pb can also form chelation complexes with certain active compounds, reducing their bioavailability. For example, Pb^2+^ binds with flavonoids to form insoluble complexes, thereby reducing their biological activity and preventing therapeutic doses from achieving their intended effects [59]. Kisa et al. [60] observed that Pb can chelate with organic acids and phenolic compounds in medicinal plants, significantly altering their concentrations. Pb stress promotes the accumulation of ursolic acid in *Prunella vulgaris*, with its content peaking at a Pb concentration of 600 mg·kg^−1^, showing a 20.5% increase compared to the untreated control. Considering the yield and quality of *Prunella vulgaris*, the critical soil Pb threshold for its cultivation is determined to be 400 mg·kg^−1^ [61]. At Pb concentrations ranging from 0 to 1000 mg·kg^−1^, Pb levels exhibited a highly significant negative linear correlation with leaf net photosynthetic rate and Soil and Plant Analysis Development (SPAD) values, while showing a significant positive linear correlation with total saponin content in *Panax ginseng* roots. This indicates that the responses of leaf photosynthesis and root secondary metabolism to Pb stress are inversely related. High concentrations of Pb stress inhibit the photosynthetic capacity of *Panax ginseng* leaves but promote the secondary metabolic processes in the roots [62]. Under high-concentration Pb treatment, the secondary metabolites (total phenolic and flavonoids) and several key enzymes involved in secondary metabolism (shikimate dehydrogenase, phenylalanine ammonia-lyase and polyphenol oxidase) were found to increase in *Vallisneria natans* [63]. Meanwhile, the increase in calcium, magnesium and iron content and the decrease in phosphorus, potassium and manganese content were detected in leaves of *V. natans* under Pb stress, while no significant changes were detected in copper and zinc concentration. These results suggest that nutrient uptake and secondary metabolism were actively regulated by *V. natans* plants in response to Pb stress. *Amaranthus spinosus* is more sensitive to Cd than Pb, and accumulates Pb and Cd primarily in the roots; Cd is more bioconcentrated and translocated compared to Pb [64]. For fennel (*Foeniculum vulgare* L.), Pb exposure significantly reduced the content of trans-anethole and oxygenated compounds, while the levels of limonene and monoterpenes increased significantly [65]. These changes directly affect the pharmacological efficacy and safety of medicinal plants.

To conclude, the presence of heavy metals such as Cd, As, and Pb generally disrupts the synthesis and accumulation of active compounds in medicinal plants, thereby diminishing their therapeutic efficacy and overall quality. However, different trends may emerge depending on the type of medicinal plant, the medicinal part, the concentration, and the specific heavy metal involved. Therefore, it highlights the importance of monitoring medicinal part quality and controlling heavy metal pollution to ensure the safety and effectiveness of medicinal plants.

### 2.3. Accumulation of Toxic and Hazardous Substances in Medicinal Plants Induced by Heavy Metals and Their Risk Assessment

The accumulation of heavy metals in medicinal plants is also a complex and critical issue influenced by multiple factors, including the type of heavy metals, soil conditions, plant species, and the specific plant parts used for medicinal purposes. Risk assessment, as an effective tool for scientifically controlling hazardous substance residues, plays a crucial role in ensuring the safety of medicinal plants [65]. A comprehensive understanding of heavy metal levels in soil and the absorption and enrichment capacities of medicinal plants enables accurate evaluation of exogenous hazardous substance risks in medicinal materials.

Firstly, different heavy metals pose varying levels of hazard indices and carcinogenic risks to medicinal plants. For example, studies assessing the carcinogenic and non-carcinogenic potential of toxic elements in *Codonopsis pilosula* revealed that the average content of nine toxic elements ranked as Al > Mn > Cu > Cr > Ni > Pb > Cd > Hg > As [66]. This study elucidated the distribution and characteristics of these elements. Cd primarily exerts its toxic effects through bioaccumulation and metabolic interference [67], while As induces extensive physiological and genotoxic effects [68]. Pb disrupts plant structure and function and induces oxidative stress [69]. Notably, even at equivalent concentrations, the risks associated with heavy metals vary significantly due to differences in their chemical forms and bioavailability.

Secondly, soil quality, as the foundation for medicinal plant growth, directly impacts the safety and quality of medicinal materials. Heavy metal pollution alters soil properties, such as causing pH imbalance and reducing organic matter content [70]. More critically, heavy metals inhibit microbial reproduction [71] and enzyme activity [72] in soil, leading to a decline in soil fertility, which negatively affects medicinal plant growth and quality.

Different medicinal plant species exhibit varying capacities to accumulate heavy metals from soil. Kong et al. [73] used ICP-MS and the Target Hazard Quotient (THQ) method to analyze heavy metal contents in *Codonopsis pilosula*, *Astragalus membranaceus*, and kelp (*Laminaria*). Their results indicated that *Astragalus membranaceus* accumulated the highest heavy metal content and posed the greatest adverse effects. Similarly, Wang et al. [74] analyzed heavy metal residues in 24 commonly marketed medicinal plants and assessed their health risks. The findings revealed that Cd levels were highest in *Lilium* bulbs, As levels were highest in dried ginger, and Pb levels were highest in *Stephania tetrandra*.

Moreover, different plant parts demonstrate varying abilities to accumulate heavy metals during growth. Li et al. [75] found that the distribution of Pb and As in plants generally followed the order: roots > leaves > stems, indicating that medicinal parts derived from plant stems carried lower risks. Other studies focusing on *Panax notoginseng* demonstrated variations in heavy metal enrichment across five plant parts—main root, rhizome head, fibrous root, stem, and leaves. Notably, the hazard index (HI) values for all parts were less than 1, indicating no significant health risks [76].

Systematic risk evaluation of heavy metal accumulation in medicinal plants requires consideration of multiple interrelated dimensions rather than isolated case studies. Effective assessment should take into account the toxicological weight of individual metals, the capacity of different plant species to absorb and translocate these elements, and the heterogeneous distribution of metals across specific medicinal parts such as roots, stems, and leaves. In addition, exposure assessments must reflect realistic patterns of medicinal plant consumption and population-specific vulnerabilities. By integrating these aspects with standardized methodologies, including the Hazard Index (HI), Target Hazard Quotient (THQ), and Target Cancer Risk (TCR), it becomes possible to compare risks across diverse metals, species, and plant parts in a coherent and structured manner. Such an approach not only improves the reliability of risk characterization but also provides a rational basis for identifying high-risk scenarios and prioritizing targeted management strategies, thereby offering essential guidance for subsequent measures aimed at reducing heavy metal contamination and safeguarding the quality of medicinal plants.

In summary, the accumulation and residual presence of heavy metals in medicinal plants are highly complex and typically exist at trace levels, but their potential risks to human health should not be underestimated. Factors such as metal properties, soil conditions, plant species, and the medicinal parts used significantly influence heavy metal accumulation. These accumulated metals may enter the human food chain, leading to long-term health issues, including neurological disorders, kidney damage, developmental impairments, and an increased risk of chronic diseases. Building upon a systematic evaluation, appropriate management measures (such as selecting low-accumulation varieties, avoiding high-contamination regions, and choosing planting sites based on the impact of heavy metals on medicinal parts), together with continuous monitoring and stringent quality control, are essential to mitigate contamination risks. These coordinated actions will ensure the quality and safety of medicinal plants, safeguard public health, and support the sustainable development of modern medicine.

## 3. Mechanisms of Heavy Metal Effects on Rhizosphere Microorganisms in Medicinal Plants

**Figure 2:**
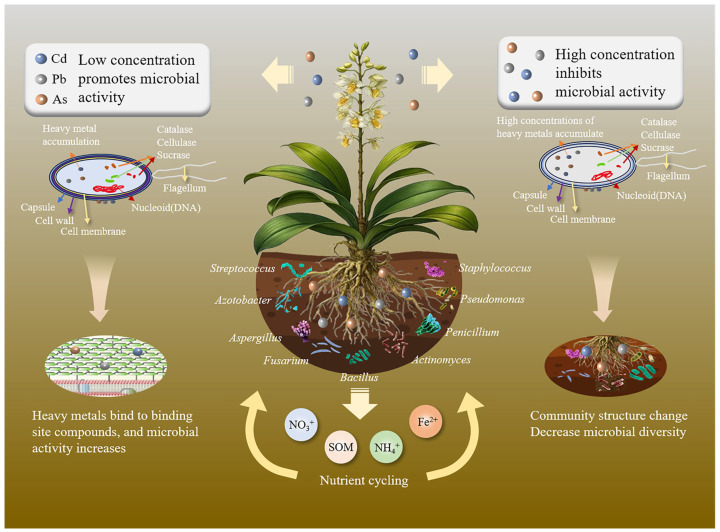
Effects of heavy metals on rhizosphere microbial communities and their role in medicinal plant quality. High concentrations of heavy metals suppress the growth and metabolic activity of rhizosphere microorganisms, alter microbial community composition and functional potential, and reduce their beneficial interactions with plant roots, which hampers plant growth and decreases the overall quality of medicinal plants.

In recent years, it has been widely recognized that rhizosphere microorganisms play an important role in plant growth and development, physiological processes, and ecological adaptation. The presence of heavy metals not only disrupts the metabolic functions of microorganisms but also alters the structure of rhizosphere microbial communities, increasing the abundance of resistant species while reducing sensitive ones [77]. These changes lead to a decline in microbial diversity and a reduction in ecological functions, particularly in soil nutrient cycling and the maintenance of medicinal plant health [78]. The current mechanisms of these effects are illustrated in Figure 2.

### 3.1. Effects of Heavy Metals on Metabolic Activities of Rhizosphere Microorganisms

#### 3.1.1. Mechanisms of Inhibition and Activation of Microbial Metabolic Pathways

The toxicity of heavy metals such as Cd and Pb in microorganisms is primarily due to their interaction with sulfur-containing enzymes, leading to enzyme inactivation. For example, hydrogen sulfide synthase and glutathione transferase, which have thiol (-SH) groups at their active sites, form stable complexes with Cd and Pb, thereby disrupting protein structure and function [79]. Duan et al. [80] found that carbon, nitrogen, and phosphorus absorption activities in rhizosphere soil were significantly higher than in non-rhizosphere soil. Under heavy metal stress, microbial carbon limitation increased while phosphorus limitation decreased, particularly in rhizosphere soils. For As(V), its structural similarity to phosphate allows it to enter the phosphorylation pathways by replacing phosphate groups, disrupting key biochemical processes such as ATP synthesis [81].

Despite the inhibitory effects of heavy metals on specific metabolic pathways, some microorganisms can activate certain metabolic mechanisms under heavy metal stress to enhance their survival. For instance, *Pseudomonas aeruginosa* can upregulate adaptive mechanisms, such as ion pumps that accelerate metal ion efflux [82], or modify lipopolysaccharide structures in the cell wall to reduce metal ion permeability [83]. These strategies enable bacteria to mitigate or evade heavy metal toxicity, allowing them to survive and propagate in contaminated environments.

#### 3.1.2. Microbial Adaptation and Gene Expression Regulation Under Heavy Metal Stress

Under heavy metal pollution, certain microorganisms can adapt and develop tolerance or resistance to heavy metals. Noor et al. [84] demonstrated that heavy metal stress activates microbial detoxification mechanisms, including the synthesis of heat shock proteins to maintain normal physiological functions and the reactivation of oxidized proteins to counter heavy metal stress. Molina et al. [85] reported that microorganisms can adapt to heavy metal stress through multiple mechanisms, such as redox processes, cellular metabolism adjustments, and intracellular ion methylation. Microbes can also regulate internal concentrations of toxic metals through active efflux and secretion mechanisms, as well as passive uptake mediated by cell wall components, proteins, and extracellular polysaccharides [86,87]. Ardissone et al. [88] found that Cd exposure inhibits methyltransferase activity, leading to DNA damage in bacteria. In response, microorganisms upregulate the expression of DNA repair proteins and ribosomal proteins to counteract heavy metal stress. These adaptations reflect the genetic and metabolic flexibility of microorganisms, enabling them to survive and maintain ecological functionality under heavy metal-contaminated conditions.

### 3.2. Effects of Heavy Metals on Rhizosphere Microbial Community Structure and Function

#### 3.2.1. Changes in Rhizosphere Microbial Community Structure

Current research demonstrates that heavy metal contamination significantly impacts the structure and function of microbial communities, and this holds true for the rhizosphere of plants. In contaminated soils, Salam et al. [89] observed that the phylum *Proteobacteria* dominated microbial communities in tropical agricultural soils, indicating that such microorganisms exhibit a high degree of adaptability to heavy metal stress. Quantitative studies have shown that heavy metal contamination significantly reduces microbial diversity indices, such as the Shannon diversity index, within rhizosphere soils [90]. For example, higher concentrations of cadmium can cause significant decreases in the Shannon and Simpson indices, potentially leading to microbial community simplification [91]. This trend has been observed across various contaminated ecosystems, with zinc (Zn) and Pb showing similar inhibitory effects on microbial diversity at concentrations above 50 mg·kg^−1^ and 100 mg·kg^−1^, respectively [92,93]. These quantitative metrics highlight the cascading effects of heavy metals on microbial community structure and their associated ecological functions. In environments with elevated heavy metal concentrations or insufficient resistance mechanisms, microbial genera such as *Pseudomonas* and *Enterobacter* are typically more sensitive to heavy metals [92] and may exhibit inhibited growth or reduced abundance. Conversely, metal-tolerant fungi such as *Aspergillus* and *Penicillium* [94], along with certain bacteria like *Staphylococcus* and *Bacillus* [95], display a relative insensitivity to heavy metal contamination. These microorganisms tend to increase in abundance under heavy metal stress, becoming dominant species within the rhizosphere microbial community and reshaping the overall community structure. Table 3 provides a consolidated overview of the relevant studies.

#### 3.2.2. Decreased Microbial Diversity and Its Ecological Consequences

Microbial diversity refers to the variation among microbial species, including their physiological functions, cellular composition, and genetic material [100]. Under heavy metal stress, microbial diversity metrics, such as the Shannon index and Simpson index, generally decrease, which may render microbial communities less stable and weaken their ability to sustain essential ecological processes [101]. These changes directly impair the nutrient cycling processes and weaken the ecological safety of medicinal plant production systems. Researchers have employed various techniques, such as phospholipid fatty acid (PLFA) profiling [102], PCR-DGGE, and BIOLOG microplate assays [101], to investigate the effects of heavy metal contamination on microbial diversity. These studies consistently reveal that microbial diversity decreases as heavy metal concentrations increase. Qian et al. [93] reported that high concentrations of heavy metals significantly reduced bacterial species richness and genetic diversity in soils, resulting in potentially negative impacts on soil ecosystem functions. Related studies have shown that heavy metal stress alters the composition of dominant rhizosphere microorganisms [103], leading to a decline in functional diversity. This reduction in microbial diversity may negatively affect the therapeutic efficacy of medicinal plants and the overall health of the ecosystem. These changes are both environmentally and ecologically important, since microbial bioremediation has emerged as a promising strategy to reduce the concentration of heavy metals in the environment due to the demonstrated ability of microorganisms, especially bacteria, to sequester and transform these compounds [92]. A previous study showed a close relationship between the tolerance level exhibited by the bacteria and metal identity, with lower Minimum Inhibitory Concentration (MIC) values found for cadmium and lead, while resistance to arsenic was widespread and significantly higher [92]. An analysis of common genera, including *Agrobacterium*, *Bacillus*, *Klebsiella*, *Enterobacter*, *Microbacterium*, *Pseudomonas*, *Rhodococcus*, and *Mesorhizobium*, revealed varying tolerance levels and highlighted biochemical and molecular mechanisms tied to plant growth promotion and resistance genes in *cad* and *ars* operons. Notably, *Klebsiella* and *Enterobacter* showed the highest cadmium and lead tolerance, effectively mitigating plant growth inhibition under toxic metal concentrations. These findings position them as prime candidates for bioremediation and bacteria-assisted phytoremediation in soils contaminated with arsenic, cadmium, and lead [92].

#### 3.2.3. Effects of Heavy Metals on Soil Nutrient Cycling Functions

Microorganisms play a pivotal role in maintaining soil fertility and ensuring sustainable production by acting as “regulators” of nutrient cycling. They control the direction, compound types, and exchange flux of soil nutrient cycles [90]. For instance, nitrogen-fixing microorganisms convert atmospheric nitrogen into ammonium, which can be readily absorbed by plants [91]. However, the presence of Cd inhibits the activity of nitrogen-fixing microorganisms, thereby reducing soil nitrogen availability, which in turn impairs soil fertility and hinders the growth of medicinal plants [104]. Furthermore, reductions in microbial diversity, as reflected by declines in the Shannon index, directly impair soil enzymatic activities critical for nutrient cycling. For instance, Cd exposure decreases soil urease activity by 40–60%, thereby disrupting nitrogen cycling processes [105]. Similarly, the inhibitory effects of heavy metals on phosphatase activity markedly reduce phosphorus availability, which is associated with shifts in microbial alpha diversity metrics. Although functional redundancy may partially buffer the impact of community simplification on ecosystem functions, the sustained decline in key enzymatic activities involved in nitrogen and phosphorus cycling suggests that functional thresholds may be exceeded, thereby weakening soil fertility and limiting nutrient supply for medicinal plants, ultimately affecting their quality and pharmacological efficacy [104,105]. The reduced microbial resilience and disrupted nutrient cycling under heavy metal contamination pose significant threats to the ecological safety of medicinal plant production systems, particularly in regions experiencing high levels of anthropogenic pollution [99,105]. These findings emphasize the importance of protecting microbial diversity in polluted soils to ensure the long-term stability of medicinal plant ecosystems (Figure 2).

### 3.3. Linking Heavy Metal-Induced Rhizosphere Microbial Shifts to the Biosynthesis of Active Compounds in Medicinal Plants

Recent research demonstrates that heavy metal stress alters rhizosphere metabolite profiles (e.g., organic acids, sugars) and the bioavailability of metals, thereby driving microbial community restructuring. In turn, the restructured microbiota—including rhizosphere bacteria, arbuscular mycorrhizal fungi (AMF), and other symbiotic fungi—can influence host plant nutrient status, metal translocation, and antioxidant metabolism through the secretion of organic acids, production of siderophores, regulation of phytohormones, and induction of antioxidant responses, ultimately affecting secondary metabolic pathways and the synthesis of pharmacologically active constituents [25,93]. Moreover, inoculation with AMF or plant growth-promoting microbes has been shown to regulate functional genes related to nutrient cycling (e.g., N, P) and metal tolerance/transport, as well as to enhance microbial network complexity and stability, thereby mitigating the adverse effects of metal stress on plant physiology and metabolism. Such mechanisms have been reported in several medicinal plant systems and have been proposed as promising approaches to improve the quality of medicinal materials [77,106]. Based on the above evidence, we emphasize that future research should integrate rhizosphere microbiome and metabolomics analyses with quantitative profiling of medicinal plant metabolites, in order to further elucidate the causal relationships among rhizosphere microbial community shifts, heavy metal stress, and the biosynthesis of bioactive constituents.

## 4. Conclusions and Future Prospects

**Figure 3:**
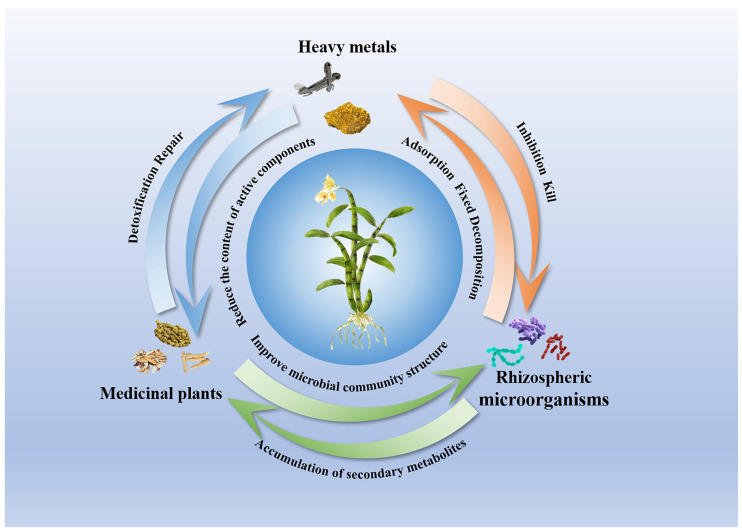
Interactions among heavy metals, medicinal plants, and rhizosphere microorganisms: A dynamic ecological system. Heavy metals accumulate in soils and plant tissues, inducing stress responses in medicinal plants. Rhizosphere microbial communities mediate plant responses by modulating nutrient availability, detoxification, and stress resilience, and the complex interplay among these three components directly affects medicinal plant quality and safety.

We illustrate the intricate relationships among heavy metals, medicinal plants, and rhizosphere microorganisms in Figure 3. The effects of heavy metal contamination on medicinal plants and their associated rhizosphere microorganisms represent a complex process involving multiple interactions and factors. Heavy metals interfere with the biological activity and metabolic functions of rhizosphere microorganisms, subsequently disrupting the structure and ecological functions of microbial communities and disturbing soil ecological balance [104]. These disruptions extend their impact to medicinal plants. Consequently, medicinal plants face dual influences from both heavy metals and rhizosphere microorganisms [107,108]. While heavy metals may inhibit plant growth and active compound synthesis, rhizosphere microorganisms play a crucial role in enhancing plant adaptability to environmental stress, promoting nutrient absorption, and increasing resistance to pathogens. Therefore, an in-depth understanding of the interactions among heavy metals, medicinal plants, and rhizosphere microorganisms is of significant importance for ensuring the quality of medicinal plants and safeguarding human health.

Heavy metal pollution affects the quality of medicinal plants and their rhizosphere microorganisms, impacting ecosystems, agriculture, and human health. Addressing this requires advancing research on interaction mechanisms, developing effective remediation technologies, and establishing robust quality and safety regulations. These efforts will support the sustainable development of the medicinal plant industry, enhance environmental protection, and safeguard public health.

(1) Enhanced monitoring and evaluation of heavy metal pollution are required

To address heavy metal contamination in medicinal plant cultivation areas, it is crucial to enhance monitoring and evaluation mechanisms for heavy metal levels in soils. This will help determine current pollution levels and trends and establish scientific thresholds and safety standards for heavy metals. Such efforts should include the formulation and refinement of maximum permissible limits for heavy metals in medicinal plants, as well as comprehensive assessments of the migration, transformation, and accumulation patterns of heavy metals in soil and plants to provide a scientific basis for pollution management.

(2) Comprehensive research on the effects of heavy metal pollution on medicinal plant quality should be undertaken

In-depth analysis of the absorption, accumulation, and translocation mechanisms of heavy metals in different medicinal plants is necessary to reveal the intrinsic relationships between heavy metal content and plant quality. Further research is needed to investigate the transformations of heavy metal forms and their bioavailability during plant growth, as well as their effects on the active compounds of medicinal plants. Such studies will provide theoretical support for quality control and risk assessment of heavy metal contamination.

(3) Rational planning and environmental management of medicinal plant cultivation areas require advancement

Rational planning of medicinal plant cultivation areas, including optimizing planting layouts, timely remediation of contaminated or heavy-metal-exceeding regions, and strengthening environmental management during the growth stages, can fundamentally reduce the impact of heavy metals on plant quality. Additionally, research on the effects of different planting models on the quality of medicinal plants and their rhizosphere microorganisms can help optimize cultivation practices to minimize the absorption and accumulation of heavy metals in medicinal plants.

(4) Greater research emphasis on the effects of heavy metal pollution on microbial ecological functions is necessary

Microbial resistance to heavy metals and their role in soil remediation are key topics in environmental science. Establishing microbial indicators for heavy metal-contaminated soils and exploring their ecological functions will aid in soil quality monitoring and risk assessment. Metagenomics can reveal microbial diversity, structure, and function under heavy metal stress, while indices like Shannon and Simpson offer insights into soil health and resilience. Integrating these metrics with enzymatic activity and nutrient cycling data provides a comprehensive view of heavy metal impacts, supporting sustainable remediation strategies [104,105].

Future research on heavy metal pollution should focus on the complex relationships between medicinal plant quality, rhizosphere microorganisms, and the ecological environment. Key areas include the migration of heavy metals in soil-plant systems, their long-term effects on active compounds and therapeutic efficacy, and their impact on microbial diversity and ecological functions. Collaborative efforts across environmental science, microbiology, and systems biology are essential for developing innovative pollution management strategies. Such research will deepen our understanding of pollution’s ecological impacts, support global control efforts, and promote the sustainable use of medicinal plant resources while safeguarding ecological and human health. Future research should prioritize the integration of high-throughput sequencing technologies and metagenomic analyses to better understand microbial adaptation and resilience mechanisms under heavy metal stress. Additionally, comprehensive risk assessment models combining toxicological thresholds with microbial community health indicators will provide a robust framework for evaluating the ecological safety of medicinal plants in contaminated environments.

## Figures and Tables

**Table 1:** Regulatory Roles of Rhizosphere Microorganisms on Medicinal Plants.

Key Mechanism	Regulatory Function	Specific Role	References
Secondary Metabolite Synthesis and Plant Health Promotion	Root Exudate Regulation	Increased content of root exudates such as oxalic acid and acetic acid affects root morphology.	[16]
	Secondary Metabolite Synthesis	Nitrogen-fixing bacteria may influence hormone signal transduction pathways.	[17]
	Stress Resistance in Medicinal Plants	Inoculation with *Bacillus* improves rhizosphere soil enzyme activity.	[18]
	Disease Resistance	Actinomycetes play a crucial role in decomposing cellulose, polyphenols, and lignin.	[19]
	Plant Hormone Regulation	Microorganisms influence plant growth by producing plant hormones or hormone analogs.	[20]
	Signal Communication	Microbial elicitors stimulate signaling pathways for the synthesis of secondary metabolites in medicinal plants.	[21,22]
	Disease Pressure Regulation	Microorganisms alter the rhizosphere environment to reduce the survival space of pathogens.	[23]
Heavy Metal Detoxification and Environmental Remediation	Heavy Metal Detoxification	Microorganisms reduce heavy metal availability in soil through chelation or adsorption.	[24]
	Biodegradation	Microorganisms metabolize recalcitrant organic macromolecules into water, carbon dioxide, and less toxic compounds.	[25]
	Bioaccumulation	Microorganisms immobilize environmental pollutants through bioaccumulation processes.	[26,27]
Nutrient Acquisition and Transformation	Nutrient Absorption and Transformation	Arbuscular mycorrhizal fungi and soil bacteria mineralize organic phosphorus and produce siderophores.	[28,29]
	Growth Promotion	Produces growth-promoting substances such as indole-3-acetic acid (IAA).	[30]
	Soil Structure Improvement	Microbial extracellular polymeric substances (EPS) improve soil physical structure and increase aggregate stability.	[31]
	Water Management	Microbial extracellular polysaccharides help retain soil moisture.	[32]
	Organic Matter Degradation	Microorganisms promote the mineralization process of organic matter in soil.	[33]

**Table 2:** Comparison of heavy metal limits in medicinal plants and herbal preparations among major international pharmacopoeias.

Authority/Source	Cd	Pb	As	Scope	References
The Pharmacopoeia of the People’s Republic of China (Vol. IV, 2020)	≤1 mg·kg^−1^	≤5 mg·kg^−1^	≤2 mg·kg^−1^	Raw herbal material	[45]
WHO (1998, Quality control methods for medicinal plant materials)	≤0.3 mg·kg^−1^	≤10 mg·kg^−1^	-	Dried plant materials	[46]
ICH Q3D (R2, 2022)—adopted by EMA & USP	5 µg/day	5 µg/day	15 µg/day	Finished products, risk-based	[47]
USP <232>/<233> (aligned with ICH Q3D)	5 µg/day	5 µg/day	15 µg/day	Finished drug products	[48]

**Table 3:** Studies on rhizosphere microbial communities associated with medicinal plants under heavy metal conditions.

Direction of Interaction	Specific Impact	References
Rhizosphere Organisms → Heavy Metals	The study showed two ecotypes recruited distinct rhizosphere microbiomes: The accumulator ecotype (AE) used diverse microbes (e.g., *Flavobacterium*) to promote growth and Pb uptake, while the non-accumulator ecotype (NAE) used specialized microbes (e.g., *Pseudomonas*) to reduce Pb stress and limit uptake. This was the first evidence that plants adapt to heavy metal stress through microbial ecological assembly.	[24]
	Metagenomic analysis showed that arbuscular mycorrhizal fungi (AMF) modified rhizosphere microbial gene expression in *Iris tectorum’s*., upregulating Cr detoxification and stress-response genes, thus enhancing plant resistance to chromium stress. This reveals microbial regulation of community function as a key mechanism of plant tolerance.	[77]
	Both *Rhizobium panacihumi* in *Ginseng* and Al stress-enriched bacteria (*Bacillus*, *Pseudomonas*) in *Ginger* enhanced Al tolerance by promoting growth, regulating metabolites and antioxidant genes, reducing ROS, and alleviating toxicity.	[96,97]
	Application of microbial inoculants (MI) and “garbage enzyme” (GE) reduced Cd in *S. miltiorrhiza* roots and altered rhizosphere microbes, increasing taxa like *Brevundimonas*, *Microbacterium*, *Cupriavidus*, and *Aspergillus*. The combined MIGE (MI+GE) treatment showed the strongest Cd reduction.	[98]
Heavy Metals → Rhizosphere Organisms	Heavy metal stress constrains microbial metabolic capacity, alters soil ecoenzymatic stoichiometry, and causes pronounced C and P limitations in the rhizosphere.	[80]
	Cadmium stress markedly alters soil microbial communities, enriching diverse heavy metals resistance and antioxidant genes that strengthen the resistome, while simultaneously causing the loss of certain species and functional genes involved in carbon and nitrogen cycling, thereby reducing ecological metabolic diversity.	[89]
	Cd and other heavy metals markedly diminish microbial diversity (e.g., Shannon index), alter relative abundance, and restructure community composition. Heavy metals act as strong environmental filters, enriching tolerant taxa (e.g., *Actinobacteria*, *Proteobacteria*) while suppressing sensitive groups (e.g., *Acidobacteria*), driving directional succession.	[93,99]

## Data Availability

No new data were created or analyzed in this study. Data sharing is not applicable to this article.
